# Intelligent-Reflecting-Surface-Assisted Single-Input Single-Output Secure Transmission: A Joint Multiplicative Perturbation and Constructive Reflection Perspective

**DOI:** 10.3390/e26100849

**Published:** 2024-10-08

**Authors:** Chaowen Liu, Anling Zeng, Fei Yu, Zhengmin Shi, Mingyang Liu, Boyang Liu

**Affiliations:** 1 School of Communication and Information Engineering, Xi’an University of Posts and Telecommunications, Xi’an 710121, China; zenganling@stu.xupt.edu.cn (A.Z.); yf@stu.xupt.edu.cn (F.Y.); shizhengmin@stu.xupt.edu.cn (Z.S.); liuboyang@xupt.edu.cn (B.L.); 2 China Academy of Space Technology Xi’an, Xi’an 710000, China; lmy19881228@126.com

**Keywords:** intelligent reflecting surface (IRS), single-input single-output (SISO), physical-layer security (PLS), joint perturbation and reflection (JPR) design, performance analysis

## Abstract

Due to the inherent broadcasting nature and openness of wireless transmission channels, wireless communication systems are vulnerable to the eavesdropping of malicious attackers and usually encounter undesirable situations of information leakage. The problem may be more serious when a passive eavesdropping device is directly connected to the transmitter of a single-input single-output (SISO) system. To deal with this urgent situation, a novel IRS-assisted physical-layer secure transmission scheme based on joint transmitter perturbation and IRS reflection (JPR) is proposed, such that the secrecy of wireless SISO systems can be comprehensively guaranteed regardless of whether the reflection-based jamming from the IRS to the eavesdropper is blocked or not. Moreover, to develop a trade-off between the achievable performance and implementation complexity, we propose both element-wise and group-wise reflected perturbation alignment (ERPA/GRPA)-based IRS reflection strategies, respectively. In order to evaluate the achievable performance, we analyze the ergodic secrecy rate (ESR) and secrecy outage probability (SOP) of the SISO secure systems with the ERPA/GRPA-based JPRs, respectively. Finally, by characterizing the simulated and numerical ESR and SOP performance results, our proposed scheme is compared with the benchmark scheme of random phase-based reflection, which strongly demonstrates the effectiveness of our proposed scheme.

## 1. Introduction

The proliferation of fifth-generation mobile communication technology (5G) has ushered the Internet of Things (IoT) into a new era, which can be characterized by an increasing number of IoT devices endowed with sensing, computing, and wireless communicating capabilities [1]. Typically, sensor nodes transmit perceived data to a central cloud server or a wireless access point. However, due to the broadcast nature of electromagnetic signals and the inherent openness of wireless channels, these systems are constantly vulnerable to eavesdropping, potentially leading to the leakage of sensitive information. Consequently, ensuring the security of wireless transmission systems has become an urgent challenge [2,3]. While traditional upper-layer encryptions play critical roles in safeguarding data transmission, their high implementation complexity may prevent them from being adopted by low-computing-resource IoT systems [4]. In recent years, physical-layer security (PLS) has garnered significant attention due to its ability to capitalize on the unique characteristics of wireless channels and diminish the signal quality received by malicious eavesdroppers with a low implementation complexity [5]. Most of the existing PLS techniques enhance the security and reliability of communication systems by leveraging advanced signal-processing techniques including physical-layer encryption, artificial jamming, and directional transmissions [6,7,8,9].

Recently, the IRS has garnered widespread attention for its capability to adaptively configure wireless transmission environments [10]. The IRS, also known as the intelligent reflective array, consists of a surface with a large number of tiny reflective elements that can intelligently modify the phases and amplitudes of incident electromagnetic waves and control them precisely [11,12,13]. Specifically, the wireless environment can be dynamically adjusted in real time to enhance the sensing reliability, transmission efficiency, and information secrecy [14]. These advantages in all these areas make the IRS a promising technology for improving transmission coverage and enabling a high spectrum and energy efficiency in both existing and future wireless networks. For instance, IRSs have been utilized to enable enhanced mobile edge computing (MEC) via improving the offloading efficiency [15], to bolster the radar performance and broaden communication coverage in integrated sensing and communication (ISAC) systems [16], and to enhance the overall efficiency in backscatter communication (BC) systems [17]. Moreover, IRS-assisted PLS techniques have been proposed to improve communication security by intelligently adjusting the phase shifts of the IRS so as to maximize the difference between the received signal-to-noise ratios (SNRs) at the intended receiver and potential eavesdroppers [18,19,20].

There have been many research works dealing with IRS-assisted PLS in wireless communication systems with single-input single-output (SISO), multiple-input single-output (MISO), and multiple-input multiple-output (MIMO) configurations [13,21,22,23,24,25,26,27,28,29,30,31,32]. Specifically, for IRS-assisted SISO communication, the authors of [21] examined the system secrecy performance in the presence of an eavesdropping user. In [22], the authors proposed to maximize the system secrecy rate by optimizing the passive beamforming matrix at the IRS. In [13], the IRS-aided wireless powered communication (WPC) system is studied in the presence of a passive eavesdropper, where three secure IRS-WPC modes are proposed based on the location of the IRS, and the asymptotic ergodic secrecy capacity of each mode is analyzed. The authors of [23] investigated the enhancement of PLS in smart grid communications through the application of the IRS, focusing on evaluating the positive impact of the IRS on the system secrecy outage probability (SOP) and ergodic secrecy rate (ESR). For the IRS-assisted MISO configuration, the authors of [24] proposed to jointly optimize the transmit active beamforming, IRS passive beamforming, and cooperative jamming matrices, so as to achieve the optimal system secrecy rate. In [25], a secure transmission strategy was deliberated, where the beamforming at Alice is designed to be aligned with the estimated Alice-to-Bob channel and the IRS is deployed to modulate the received confidential signal into the jamming signal by adjusting the reflection coefficients strategically. In [26], the authors investigated an IRS-assisted secure wireless network consisting of multiple users and a multiantenna base station, in which the uplink secure transmission of a specific user is guaranteed by randomly located friendly jammers. In [27], the secrecy performance of an IRS-aided communication system in the presence of spatially random UAV eavesdroppers was analyzed. In [28], the authors investigated the PLS of an IRS-assisted multiantenna communication system in the presence of spatially random eavesdroppers. Based on the theory of stochastic geometry, the closed-form expressions for the system SOP and ESR were obtained. Furthermore, for the IRS-assisted MIMO configuration, the authors of [29,30] further proposed to employ artificial noise (AN) schemes to enhance the system security performance. To demonstrate the technical superiority, the authors of [31] proposed an IRS-assisted downlink secure transmission framework against a multiantenna eavesdropper. In [32], the authors derived the secrecy performance result over an IRS-assisted MIMO wiretapping channel using the central limit theorem (CLT) and random matrix theory.

Based on the related works, one can conclude that the implementation of IRS-assisted PLS techniques relies primarily on augmenting the difference in signal quality between the legitimate receiver and eavesdropper. This can be typically achieved via utilizing either individual or joint beamforming, AN, and collaborative jamming techniques. However, the beamforming techniques request the transmitter to be equipped with multiple antennas, which may be forbade to be implemented with resource-restricted users. The utilization of AN or collaborative interference methods often results in increased communication power, time-slots and hardware consumptions. Due to these, despite extensive research conducted, the related study on guaranteeing single-antenna-based secure transmission is still in its infancy. In other words, there remains a research gap in the IRS-assisted SISO secure communications. To be specific, how to comprehensively enhance the transmission security in an SISO communication scenario without or with only few additional resource consumptions remains an open issue. These in all motivate our work.

Against the above background, this paper introduces and investigates a novel IRS-enabled SISO secure transmission system. In this system, the transmitter sends a perturbation signal alongside the information signal to guarantee the system’s secrecy without pending any resource consumptions. Further, we introduce a joint transmitter perturbation and IRS reflection (JPR) approach so as to enhance the security of SISO transmissions, namely maximizing the transmission rate of the legitimate receiver while decreasing the eavesdropping rate as much as possible. To demonstrate the comprehensiveness of our proposal in guaranteeing secrecy, we consider two typical eavesdropping scenarios based on the fact that the IRS is occasionally connected to the malicious passive eavesdropper or not. The main contributions of this paper can be summarized as follows.

- We propose an IRS-assisted SISO secure transmission system, where the transmitter sends a scrambling signal alongside the information signal to comprehensively guarantee the system’s secrecy. To thoroughly evaluate the system’s capability of anti-wiretapping, we consider two eavesdropping scenarios, where a malicious passive eavesdropper possesses direct links with and without the IRS, respectively.
- We introduce a joint transmitter perturbation and IRS reflection (JPR) approach so as to enhance the system’s secrecy. The JPR approach is framed based on the reflection perturbation alignment (RPA) strategy, so as to eliminate phase distortions at the legitimate receiver, while simultaneously constraining the eavesdropper’s ability to receive the confidential signals. Additionally, we also present IRS element/group-wise RPA (ERPA/GRPA)-based JPRs to attain different extents of trade-offs between the system implementation complexity and achievable performance.
- The secrecy performance results of the IRS-assisted SISO secure transmission system are analyzed and evaluated. Specifically, we derive the closed-form or semi-closed-form ESR and SOP under different JPR strategies and distinct eavesdropping scenarios. Additionally, the simulated and numerical results of ESR and SOP are characterized and investigated so as to substantiate the theoretical derivations and reveal the technical superiorities of our proposed secure transmission.

The rest of the article is organized as follows. Section 2 presents the proposed system model and two typical eavesdropping scenarios. In Section 3, two IRS reflections-based JPR designs are proposed. Section 4 derives the theoretical ESR and SOP with different JPR designs. In Section 5, simulated and theoretical performance results are demonstrated to illustrate the advantages of the proposed schemes. Section 6 concludes with the summary of this paper.

*Notations*: Bold uppercase letters indicate matrices, while bold lowercase letters indicate vectors. $C^{m\times n}$ denotes the space of (m×n) complex-valued matrices. $A_{m\times n}$ represents a matrix of *m* rows and *n* columns. $j=\sqrt{-1}$ is the imaginary unit. E[x] represents the expectation operator of variable *x*. $\left|\cdot \right|$ denotes the norm of a complex scalar. diag[·] denotes the diagonalization operator. ${\left[x\right]}^{+}=\max\left\{0,x\right\}$. $M_{\lambda ,\mu }\left(x\right)$ is the Whittaker function of *x*, and $I_{x}\left(\cdot \right)$ is the first-class Bessel correction function of the independent variable *x*.

## 2. System Model

**Figure 1:**
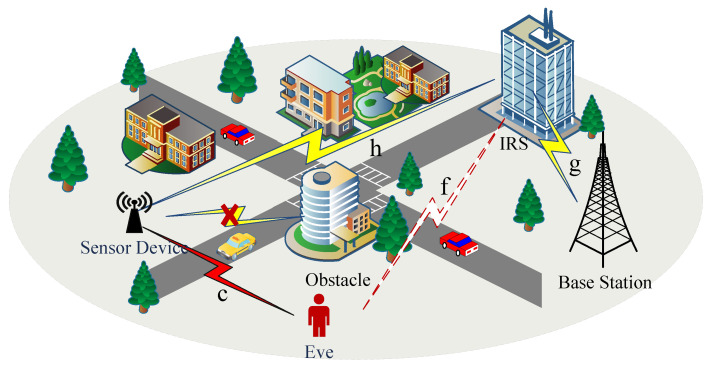
An IRS-aided SISO secure transmission system.

As illustrated in Figure 1, we consider an IRS-aided secure transmission system, wherein a legitimate source device (SD) transmits its confidential information to a legitimate base station (BS) with the assistance of an IRS having *N* passive reflective elements, while an eavesdropper (Eve) passively tries to overhear the transmitted information from both SD and IRS. For simplicity, *N* is supposed to be a non-negative integer power of 2. In this paper, we assume all the communication nodes of SD, BS and Eve are equipped with single antenna and operate in the half-duplex mode. Moreover, there is no direct link between SD and BS due to the heavy path loss and shadowing. In practice, the reflection coefficients of the IRS are regulated by SD via a controller connected between them. Based on the channel state information (CSI) acquired, the IRS can intelligently regulate the phase and amplitude of the confidential signals delivered from SD to BS in a real-time manner [31]. Additionally, it is also assumed that the channels associated with legitimate and wiretap links are reciprocal, quasi-static, and subject to independent and identically distributed (i.i.d.) Rayleigh fading. The channels from SD to the IRS, the IRS to BS, SD to Eve, and that from the IRS to Eve are denoted as $h={\left[h_{1},h_{2},\ldots ,h_{l},\ldots ,h_{N}\right]}^{T}\in C^{N\times 1}$, $g=\left[g_{1},g_{2},\ldots ,g_{l},\ldots ,g_{N}\right]\in C^{1\times N}$, $c\in C^{1\times 1}$ and $f=\left[f_{1},f_{2},\ldots ,f_{l},\ldots ,f_{N}\right]\in C^{1\times N}$, respectively. The reflection coefficients matrix of IRS is represented as a diagonal matrix $\Phi =\mathrm{diag}[e^{-j\varphi_{1}},e^{-j\varphi_{2}}$$,\ldots ,e^{-j\varphi_{l}},\ldots ,e^{-j\varphi_{N}}]$ with the phase shift component of the *l*-th IRS element $\varphi_{l}$ being arbitrarily selected from the continuous interval of (0,2π], where l∈{1,2,…,N}.

In this paper, we assume that the CSI between SD and BS is obtained via transmitting pilot signals. Specifically, all the array elements of the IRS are first designated with the same reflecting amplitude of 1 and phase shift of 0. Then, BS sends commonly shared pilot signals to SD. As a result, based on the channel reciprocity, SD can obtain the CSI of the cascade channel between SD and BS. In addition, we assume that Eve can only know the CSI of the direct channel between SD and Eve, but it cannot obtain the CSI of the IRS-involved cascade wiretapping channel (The assumption regarding Eve’s ability to acquire channel information is quite stringent. Due to occlusions between Eve and the IRS, their connection is random, which introduces uncertainty in Eve’s channel estimation of the IRS–Eve link when using pilot signals. To demonstrate the effectiveness of the proposed scheme, we assume that Eve can only access CSI about the direct SD–Eve channel and cannot obtain CSI about the cascade channel). According to the secure mechanism raised in this paper, it is obvious that the equivalent phase shifts of the IRS observed by Eve are kept disrupted. Thus, it is difficult for Eve to obtain the CSI reflected by the IRS. In addition, we can apply a private channel estimation method such that the pilot signals are sent by SD, and resultantly the legitimate CSI can be protected from being completely leaked [33].

According to the above-mentioned, in the proposed system, SD emits a random perturbation signal to Eve and BS, which can be represented as

(1) $$v_{\mathrm{pk}}=e^{-jQ}s_{k},$$

where $s_{k}$ is the confidential signal and satisfies $E[|s_{k}{|}^{2}]=1$. Q∈(0,2π] is the phase shift of a complex-valued multiplicative perturbation factor with a normalized power amplification component. Then, the legitimate signal observed at BS can be expressed as

(2) $$\begin{array}{c} y_{b}=g\Phi hv_{\mathrm{pk}}+n_{b}, \end{array}$$

and the instantaneous signal-to-noise ratio (SNR) at BS can be given by

(3) $$\gamma_{b}=\frac{|g\Phi h{|}^{2}}{\sigma_{b}^{2}},$$

where $n_{b}$ denotes the complex Gaussian noise at BS with a zero mean and variance of $\sigma_{b}^{2}$.

In order to achieve significantly enhanced confidentiality, it is required to maximize the achievable SNR at the legitimate receiver while suppressing the SNR at Eve. For accomplishing this, we propose the JPR that co-designs the transmitter perturbation and IRS reflections. By doing so, the phases of the signal observations obtained by BS are fully compensated. However, the phases of the signals observed by Eve are randomly and independently updated between any adjacent two symbol durations, since the reflected signal received by Eve is highly dislocated in the symbol rate. As a result, Eve’s detection of confidential signals emitted by SD may be severely deteriorated. On this basis, the equivalent cascaded channel of the SD–IRS–Eve link (if it is not blocked) can be considered as an interference link.

From the above-elaborated perspective, it is reasonable to consider and investigate two eavesdropping scenarios: (1) the ideal wiretap scenario (IWS), where Eve has a direct link with SD and the transmission link from the IRS to Eve is blocked; and (2) the worst wiretap scenario (WWS), where Eve possesses transmission links to both SD and the IRS. Accordingly, the signal observations achieved by Eve in the scenarios of IWS and WWS can be, respectively, represented as

(4) $$y_{e}^{I}=\tilde{c}+n_{e},$$

(5) $$\begin{array}{c} \begin{array}{c} y_{e}^{W}=\tilde{c}+f\Phi hv_{\mathrm{pk}}+n_{e}, \end{array} \end{array}$$

where $\tilde{c}=cv_{\mathrm{pk}}$. $n_{e}$ denotes the complex Gaussian noise at Eve with zero mean and variances of $\sigma_{e}^{2}$. Then, the instantaneous SNR and signal-to-interference-plus-noise ratio (SINR) attained by Eve in the two eavesdropping scenarios are, respectively, formulated as

(6) $$\gamma_{e}^{I}=\frac{|\tilde{c}{|}^{2}}{\sigma_{e}^{2}},$$

(7) $$\gamma_{e}^{W}=\frac{|\tilde{c}{|}^{2}}{|f\Phi h{|}^{2}+\sigma_{e}^{2}}.$$

## 3. RPA-Based JPR Secure Transmission

The objective of the proposed RPA-based JPR is to augment the SNR/SINR discrepancy between Bob and Eve. To accomplish this, the IRS reflection strategy of RPA is proposed (In this paper, the direct connection between SD and BS is not considered. However, rendering that there exists a direct connection between SD and BS, we need to implement an improved RPA strategy to fulfill the design of secure transmission. Specifically, the perturbation phase of SD can be designed based on the instantaneous CSI of the channel between SD and BS to counteract the phase effect of the directly connected channel. Meanwhile, IRS-reflecting coefficients are designed based on the instantaneous cascaded CSI from SD to BS so as to mitigate the random perturbation phase-effects at the BS. Hence, based on our proposed methodology, the security of the IRS-assisted SISO can be comprehensively assured, no matter whether the SD is directly connected with the BS or not). The principle of RPA is that the IRS adjusts the perturbation phase of the equivalent multipath channels according to the coherent superposition principle of multipath reflections. Specifically, SD sends a confidential signal, which is masked by a multiplicative perturbation. Then, the phase-shifts of IRS elements are rectified upon utilizing the instantaneous CSI of the cascaded multipath channel from SD to BS, such that the random perturbed phases of the received signal observations are well aligned at BS. Further, the IRS element-wise RPA (ERPA) and group-wise RPA (GRPA) strategies are presented for obtaining different trade-offs between the achievable performance and implementing complexity of the system, respectively [34].

**Figure 2:**
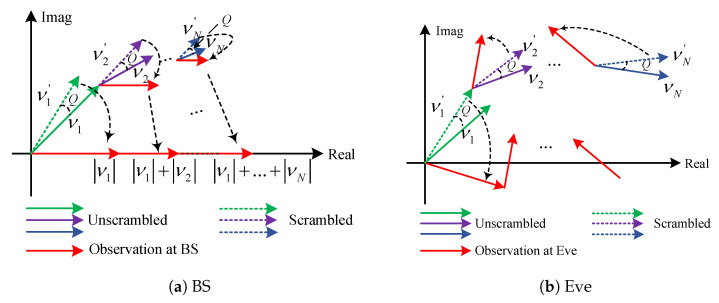
An illustration of received signal observations at BS and Eve with the RPA-based JPR secure transmissions.

To demonstrate the principle of RPA, we provide Figure 2 as an illustration of the received signal observations at BS and Eve with the RPA-based JPR secure transmissions, respectively. As shown in Figure 2, $v_{l}$ represents the cascaded channel vector from SD to BS/Eve via the *l*-th IRS reflection element, and $v_{l}^{\prime }$ represents the scrambled cascaded channel vector with the random perturbed confidential signal delivered through $v_{l}$. By adopting the RPA-based reflection, the inherent perturbation *Q* injected for secure transmission can be mitigated at BS, but it remained highly disturbed at Eve.

### 3.1. ERPA Based JPR Design

Given the ERPA is employed, the instantaneous signal observation obtained at the BS can be characterized as

(8) $$\begin{array}{c} y_{b}=e^{-jQ}s_{k}\sum_{l=1}^{N}g_{l}h_{l}e^{-j\varphi_{l}}+n_{b}. \end{array}$$

Let us define $g_{1}=\left|g_{l}\right|e^{j∠g_{l}}$ and $h_{1}=\left|h_{l}\right|e^{j∠h_{l}}$. Then, in order to maximize $\gamma_{b}$ in (3), the reflection phase shift of the *l*-th IRS element can be designed as

(9) $$\varphi_{l}^{E}=\left(-Q+∠h_{l}+∠g_{l}\right)\;\;\mathrm{mod}\;\;2\pi .$$

Accordingly, the instantaneous signal observation obtained by BS can be re-represented as (In this paper, the IRS reflection is implemented with continuous phase design, which is distinct from quantized discrete phase designs. With continuous phase design, our proposed IRS reflection is capable of eliminating the phase offsets of the cascaded legitimate multipath channels. In contrast, when a quantized discrete phase design is assumed, the legitimate transmission may suffer from quantization phase errors, and the corresponding secrecy performance can be deteriorated heavily. However, as the continuous phase design is the primary focus, the evaluation of the negative effects of quantization phase errors on the system secrecy is left for future exploration)

(10) $$y_{b}=s_{k}\sum_{l=1}^{N}|g_{l}\left|\right|h_{l}|+n_{b}.$$

In contrast, in the IWS scenario, since the transmission link from SD to IRS is blocked, the instantaneous signal observation obtained at Eve is represented the same as in (4).

On the other hand, in the scenario of WWS, upon substituting (9) into (5), the instantaneous signal observation obtained at Eve with the ERPA can be updated as

(11) $$y_{e}^{W}=e^{-jQ}s_{k}\left(c+\sum_{l=1}^{N}|f_{l}\left|\right|h_{l}|e^{j(∠f_{l}-∠g_{l})}\right)+n_{e}.$$

### 3.2. GRPA Based JPR Design

Given that the GRPA is exploited, all reflecting elements of the IRS are divided equally into *U* groups, each group containing A=N/U elements, where the elements in the same IRS group are assigned a common reflection coefficient. Similar to the derivation of (9), the phase shift of the *t*-th IRS element in the *u*-th group can be obtained as

(12) $$\varphi_{u,t}^{G}\overset{\Delta }{=}\left(\arg\left(\sum_{t=1}^{A}g_{t}^{u}h_{t}^{u}\right)-Q\right)\;\mathrm{mod}\;2\pi ,$$

where $g_{t}^{u}$ is the channel component from the *t*-th IRS element in the *u*-th group to BS, and $h_{t}^{u}$ is the channel component from SD to the *t*-th IRS element in the *u*-th group. Then, the instantaneous signal observation obtained by BS with the GRPA-based JPR can be represented as

(13) $$y_{b}=s_{k}\sum_{u=1}^{U}\sum_{t=1}^{A}|g_{t}^{u}\left|\right|h_{t}^{u}|e^{-j[\arg\left(\sum_{t=1}^{A}g_{t}^{u}h_{t}^{u}\right)-∠h_{t}^{u}-∠g_{t}^{u}]}.$$

Alternately, in the scenario of IWS, the instantaneous signal observation obtained at Eve is still represented as (4). By contrast, the instantaneous signal observation obtained by Eve in the scenario of WWS can be updated as

(14) $$\begin{array}{c} \begin{array}{c} y_{e}^{W}=s_{k}\left(\tilde{c}+\sum_{u=1}^{U}\sum_{t=1}^{A}|f_{t}^{u}\left|\right|h_{t}^{u}|e^{-j[\arg\left(\sum_{t=1}^{A}g_{t}^{u}h_{t}^{u}\right)-∠f_{t}^{u}-∠h_{t}^{u}]}\right)+n_{e}. \end{array} \end{array}$$

**Remark** **1.** *In our proposed design, there is no need for the IRS to acquire perfect Q so as to accomplish phase compensation at the BS. Instead, the SD employs the estimated CSI and the injected phase perturbation Q to determine and adjust the reflection coefficients of passive IRS via an IRS controller [35]. Second, if the SD can transmit to the BS directly, it may not be possible to accomplish the SISO secure transmission without IRS, as the direct transmission from SD to BS leads to the leakage of Q with highly probability. Third, rendering that the explored IRS is replaced by a DF relay, the involved wireless channels are restricted to satisfy flat fading, while the relays are supposed to be built with massive antenna-based transceive array(s). These in all make it too challenging to be implemented. Finally, when phase errors are yielded during the process of CSI estimation, the spatial diversity effect engaged with the IRS can be diminished, and hence the system secrecy can be deteriorated heavily.*

**Remark** **2.** *In our proposed JPR, the random phase perturbation is first injected at the transmitter without pending power consumptions; then, the reflection coefficients of the passive IRS are dedicatedly selected such that the overall phase scrambling is effectively eliminated at the legitimate receiver. Hence, we can conclude in theory that the proposed JPR focuses mainly on determining the IRS reflection coefficients based on the randomly specified phase perturbations. However, with only the legitimate CSIs being achieved at the SD, our proposed ERPA-based JPR can be recognized as optimal from the perspective of IRS reflection. Specifically, in the IWS scenario, with the acquired CSIs of* g *and* h*, the optimal* **Φ** *obtained via phase cancellation is consistent with the result achieved via utilizing the proposed ERPA scheme. By contrast, in the WWS scenario, due the fact that the instantaneous CSI of Eve is not available, the* **Φ** *achieved with the proposed ERPA may still be regarded as optimal, since the transmission rate at the legitimate receiver is maximized to achieve the best probable secrecy.*

**Remark** **3.** *From (8)–(14), we can observe that the SNR at BS with the ERPA-based JPR is higher than that with the GRPA-based one. For the GRPA scheme, as each subset is assigned with one common phase shift, the system complexity for accomplishing the IRS control and reflection can be decreased significantly. Hence, given that the GRPA-based JPR is utilized, the transmission reliability of the secure communication system is sacrificed to trade for the decrease of system implementing complexity. In this sense, the ERPA scheme can be recognized as an optimal IRS reflection design, while the GRPA scheme can be regarded as a low-complexity suboptimal IRS reflection design.*

## 4. Secrecy Performance Evaluation

In this section, we evaluate the impact of different eavesdropping scenarios and JPR strategies on system secrecy performance from the perspective of theoretical derivation. Specifically, the system’s achievable ESR and SOP are derived, respectively.

In theory, the ESR is the maximum secure transmission rate that the system can achieve for assuring the security of information transmission. It is an important indicator for evaluating the secrecy performance of information transmission, and it is usually taken as the difference between the ergodic transmission rates of BS and Eve, which can be formulated as [36]

(15) $$E\left[R_{s}\right]=E{\left[R_{b}-R_{e}\right]}^{+},$$

where $R_{b}$ and $R_{e}$ are the transmission rates of BS and Eve, respectively.

### 4.1. ESR Derivation

#### 4.1.1. ERPA-Based Derivation

On the basis of the SNR or SINR detailed in Section 2, the ergodic transmission rate at BS with the ERPA based JPR can be expressed as

(16) $$\begin{array}{c} \begin{array}{cc} {\bar{R}}_{b} & =E\left[R_{b}\right]=E_{g\Phi h}\left[\log_{2}\left(1+\frac{|g\Phi h{|}^{2}}{\sigma_{b}^{2}}\right)\right]. \end{array} \end{array}$$

It is obvious that a further simplification of (16) is difficult due to the complicated integrations involved in the statistical expectation. Thus, instead of seeking for the closed-form expressions directly, we derive a lower bound for ${\bar{R}}_{b}$ by introducing the Jensen’s inequality as follows.

(17) $$\begin{array}{c} \begin{array}{c} {\bar{R}}_{b}\geq \log_{2}\left(1+\frac{E_{g\Phi h}\left[|g\Phi h{|}^{2}\right]}{\sigma_{b}^{2}}\right). \end{array} \end{array}$$

From (17), one can see that by deriving the probability density function (PDF) of the variable $|g\Phi h{|}^{2}$, it is easy to obtain the ergodic transmission rate at BS with the ERPA-based JPR. After in-depth analysis, we find that when considering the Rayleigh fading channel, the variable $x=|g\Phi h{|}^{2}=|\sum_{l=1}^{N}|g_{l}\left|\right|h_{l}|{|}^{2}$ obeys a non-central chi-square distribution with degrees of freedom of 1, mean of $NE[|g_{l}||h_{l}|]=\pi N/4$, and variance of $ND[|g_{l}\left|\right|h_{l}\left|\right]=\left(16-\pi^{2}\right)N/16$. Consequently, the closed-form expectation of $|g\Phi h{|}^{2}$ can be derived as in Theorem 1.

**Theorem** **1.** *The expectation of $|g\Phi h{|}^{2}$ can be derived as follows*

(18) $$\begin{array}{c} \begin{array}{cc} E_{g\Phi h}\left[|g\Phi h{|}^{2}\right] & =\int_{0}^{\infty }xf(x;1,\frac{\pi^{2}}{16}N^{2})dx\\ \; & \;\;=\frac{e^{\frac{\pi^{2}N}{4\pi^{2}-64}}}{\sqrt{\pi N}}{\left(\frac{8}{(16-\pi^{2})N}\right)}^{-\frac{5}{4}}M_{-\frac{5}{4},-\frac{1}{4}}\left(\frac{\pi^{2}N}{2(16-\pi^{2})}\right), \end{array} \end{array}$$

where $f(x;1,\frac{\pi^{2}}{16}N^{2})$ is the PDF of $|g\Phi h{|}^{2}$.

**Proof of Theorem** **1.** See Appendix A for proof details. □

Substituting (18) into (17), the lower bound of ${\bar{R}}_{b}$ can be derived as

(19) $${\bar{R}}_{b}\geq \log_{2}\left(1+\frac{1}{\sigma_{b}^{2}}\frac{e^{\frac{\pi^{2}N}{4\pi^{2}-64}}}{\sqrt{\pi N}}{\left(\frac{8}{(16-\pi^{2})N}\right)}^{-\frac{5}{4}}M_{-\frac{5}{4},-\frac{1}{4}}\left(\frac{\pi^{2}N}{2(16-\pi^{2})}\right)\right).$$

Alternatively, in the scenario of IWS, the ergodic transmission rate at Eve can be given by

(20) $$R_{e}^{I}=\log_{2}\left(1+\gamma_{e}^{I}\right)=\log_{2}\left(1+\frac{|\tilde{c}{|}^{2}}{\sigma_{e}^{2}}\right),$$

where $\tilde{c}$ obeys the Gamma distribution $\Gamma \left(1,1\right)$ with the PDF of $f\left(\tilde{c}\right)=\exp\left(-\tilde{c}\right)$. Thus, the expectation of $|\tilde{c}{|}^{2}$ can be obtained as $E_{\tilde{c}}\left[\right|\tilde{c}{|}^{2}]=\int_{0}^{\infty }\tilde{c}f\left(\tilde{c}\right)d\tilde{c}=1$. Eventually, a lower bound on the ergodic transmission rate at Eve can be derived as

(21) $$\begin{array}{c} \begin{array}{cc} {\bar{R}}_{e}^{I} & =E\left[R_{e}^{I}\right]=E_{\tilde{c}}\left[\log_{2}\left(1+\frac{|\tilde{c}{|}^{2}}{\sigma_{e}^{2}}\right)\right]\\ \; & \geq \log_{2}\left(1+\frac{1}{\sigma_{e}^{2}}\right). \end{array} \end{array}$$

In the scenario of WWS, by utilizing Jensen’s inequality, the ergodic transmission rate at Eve can be upper bounded as

(22) $$\begin{array}{cc} {\bar{R}}_{e}^{W}= & E\left[R_{e}^{W}\right]=E_{\tilde{c},f\Phi h}\left[\log_{2}\left(1+\frac{|\tilde{c}{|}^{2}}{|f\Phi h{|}^{2}+\sigma_{e}^{2}}\right)\right]\\ & \leq \log_{2}\left(1+E_{\tilde{c},f\Phi h}\left[\frac{|\tilde{c}{|}^{2}}{|f\Phi h{|}^{2}+\sigma_{e}^{2}}\right]\right), \end{array}$$

where $|f\Phi h{|}^{2}=\left|e^{-jQ}{|}^{2}\right|\sum_{l=1}^{N}|f_{l}\left|\right|h_{l}|e^{j(∠f_{l}-∠g_{l})}{|}^{2}$. The phase $\left(∠f_{l}-∠g_{l}\right)$ obeys a uniform distribution of [0,2π). Defining $y=|f\Phi h{|}^{2}+\sigma_{e}^{2}$, and $z=|\tilde{c}{|}^{2}/\left(|f\Phi h{|}^{2}+\sigma_{e}^{2}\right)$, we know that the mean and variance of the variable y are $\sigma_{e}^{2}$ and $N^{2}$, respectively. As $|f\Phi h{|}^{2}$ follows the exponential, the PDF of *y* can be obtained as $f\left(y\right)=\exp\left(-\frac{y-\sigma_{e}^{2}}{N}\right)/N$. Moreover, it can also be shown that the variable *z* is composed of a multiplication of two independent variables. Then, based on the statistical theory of the product distribution of independent random variables, the PDF of *z* is actually a joint PDF of the two multiplied variables, and this can be given by

(23) $$f\left(z\right)=\frac{N\sigma_{e}^{2}z+\sigma_{e}^{2}+N}{{\left(Nz+1\right)}^{2}}e^{-\sigma_{e}^{2}z}.$$

Consequently, the expectation of *z* can be derived by utilizing (23), and substituting it into (22), so as to derive an upper bound on ${\bar{R}}_{e}^{W}$ as

(24) $$\begin{array}{cc} {\bar{R}}_{e}^{W} & \leq \log_{2}\left(1+N\sigma_{e}^{2}\int_{0}^{\infty }\frac{z^{2}}{{\left(Nz+1\right)}^{2}}e^{-\sigma_{e}^{2}z}dz+\left(\sigma_{e}^{2}+N\right)\int_{0}^{\infty }\frac{z}{{\left(Nz+1\right)}^{2}}e^{-\sigma_{e}^{2}z}dz\right)\\ \; & =\log_{2}\left(1-\frac{1}{N}\mathrm{Ei}\left(-\frac{1}{N\sigma_{e}^{2}}\right)e^{-\frac{1}{N\sigma_{e}^{2}}}\right), \end{array}$$

where $\mathrm{Ei}\left(x\right)=-\int_{-x}^{\infty }\frac{e^{-t}}{t}dt$. Ultimately, upon substituting (19), (21) and (24) into (15), the closed-form ESR results achieved by employing the ERPA-based JPR strategy can be obtained.

#### 4.1.2. GRPA-Based Derivation

Similar to the ERPA-based ESR analysis, we first give a simplified form of the variable $|g\Phi h{|}^{2}$ with the GRPA-based JPR as

(25) $$|g\Phi h{|}^{2}=|\sum_{u=1}^{U}\sum_{t=1}^{A}|g_{t}^{u}\left|\right|h_{t}^{u}|e^{-j[\arg\left(\sum_{t=1}^{A}g_{t}^{u}h_{t}^{u}\right)-∠h_{t}^{u}-∠g_{t}^{u}]}{|}^{2}.$$

By analyzing (25), there are redundant phases for the elements in arbitrary IRS group, but the redundant phase for each IRS group is 0. Therefore, we cannot use either the non-central or the central chi-square distribution to obtain the PDF of variable $x=|g\Phi h{|}^{2}$. To cope with this, we adopt the Gamma approximation to facilitate the forthcoming derivations.

**Theorem** **2.** *When the GRPA strategy is employed, the closed-form lower bound of ${\bar{R}}_{b}$ can be deduced as*

(26) $$\begin{array}{cc} {\bar{R}}_{b} & \geq \log_{2}\left(1+\frac{b\Gamma (a+1)}{\Gamma \left(a\right)\sigma_{b}^{2}}\right), \end{array}$$

*where Γ(x) is the Gamma function.*

**Proof of Theorem** **2.** See Appendix B for proof details. □

In the scenario of IWS, the upper bound on the ergodic transmission rate of Eve with the GRPA-based JPR is the same as the result with the ERPA-based one, and it is hence omitted here for simplicity. On the other hand, in the WWS, the phase component of $\left(\arg\left(\sum_{t=1}^{A}g_{t}^{u}h_{t}^{u}\right)-∠f_{t}^{u}-∠h_{t}^{u}\right)$ in (26) retains to be uniformly distributed within the range of [0,2π). Therefore, similar to the ERPA-based analysis, the upper bound on the ergodic transmission rate achieved by Eve can be obtained the same as that in (24).

### 4.2. SOP Derivation

The SOP represents the probability that a secure transmission scheme or system will fail or be breached. It is a statistical performance metric, which can be used to evaluate the effectiveness of the investigated wireless secure transmission scheme. In general, SOP is characterized as the occurring probability of the interruption that the secrecy rate $R_{s}$ is less than a given threshold of $R_{s}^{Th}$, i.e., $R_{s}<R_{s}^{Th}$, or the transmission rate $R_{b}$ is less than a threshold of $R_{b}^{Th}$. Accordingly, the SOP can be generally expressed as

(27) $$\begin{array}{c} \begin{array}{cc} P_{\mathrm{SO}} & =P\left\{R_{s}<R_{s}^{Th}|R_{b}\geq R_{b}^{Th}\right\}+P\left\{R_{b}<R_{b}^{Th}\right\}\\ \; & =P\{\frac{1+\gamma_{b}}{1+\gamma_{e}}<2^{R_{s}^{Th}}|\gamma_{b}\geq 2^{R_{b}^{Th}}-1\}. \end{array} \end{array}$$

In the following, we analyze the SOP achieved with the ERPA and GRPA-based JPRs, respectively. To facilitate the performance derivation, the SNR and SINR at BS and Eve for different eavesdropping scenarios are primarily expressed as

(28) $$SNR_{b}=\gamma_{b}=\frac{|g\Phi h{|}^{2}}{\sigma_{b}^{2}},$$

(29) $$SINR_{e}^{I}=\gamma_{e}^{I}=\frac{|\tilde{c}{|}^{2}}{\sigma_{e}^{2}},$$

(30) $$SINR_{e}^{W}=\gamma_{e}^{W}=\frac{|\tilde{c}{|}^{2}}{|f\Phi h{|}^{2}+\sigma_{e}^{2}}.$$

#### 4.2.1. ERPA-Based Derivation

According to (10) and (28), the SNR at BS with the ERPA-based JPR strategy is

(31) $$SNR_{b}^{E}=\frac{|\sum_{l=1}^{N}|g_{l}\left|\right|h_{l}|{|}^{2}}{\sigma_{b}^{2}},$$

where $|\sum_{l=1}^{N}|g_{l}\left|\right|h_{l}|/\sigma_{b}{|}^{2}$ obeys the non-central chi-square distribution with degrees of freedom as 1 and Gaussian random variable distribution with mean $NE\left[\frac{|g_{l}\left|\right|h_{l}|}{\sigma_{b}}\right]=\frac{\pi N}{4\sigma_{b}}$ and variance $ND\left[\frac{|g_{l}\left|\right|h_{l}|}{\sigma_{b}}\right]=\frac{16-\pi^{2}}{16\sigma_{b}^{2}}N$. Substituting the mean and variance into (A1), the PDF of the SINR at BS $f_{W}^{E}\left(\varpi \right)$ can be obtained as $f_{W}^{E}\left(\varpi \right)=f\left(\varpi ;1,\frac{\pi N}{4\sigma_{b}}\right)$. Similarly, the PDF of the SINR at Eve for two eavesdropping scenarios can be obtained as $f_{\Lambda ,I}^{E}\left(v\right)=\sigma_{e}^{2}e^{-\sigma_{e}^{2}v}$ and $f_{Z,W}^{E}\left(z\right)=\frac{N\sigma_{e}^{2}z+\sigma_{e}^{2}+N}{{\left(Nz+1\right)}^{2}}e^{-\sigma_{e}^{2}z}$, respectively. Accordingly, the ERPA-based SOP can be derived as in the following theorem, where we consider a comprehensive outage analysis in two separate cases: (1) the SOP associated with case $T_{1}$, i.e., $P_{\mathrm{SO}}^{T_{1}}$; (2) the SOP associated with case $T_{2}$, i.e., $P_{\mathrm{SO}}^{T_{2}}$. Here, $T_{1}$ is corresponding to the case for which the transmission rate threshold at BS is less than the system secrecy rate threshold, i.e., $R_{b}^{Th}<R_{s}^{Th}$. By contrast, $T_{2}$ is the case in which $R_{b}^{Th}\geq R_{s}^{Th}$.

**Theorem** **3.** *In the scenarios of IWS and WWS, the semi-closed-form results of the approximated SOPs can be, respectively, deduced as*

(32) $$\begin{array}{c} \begin{array}{c} P_{\mathrm{SO}}^{I,T_{1}}=B_{1}\left(\int_{2^{R_{b}^{Th}}-1}^{2^{R_{s}^{Th}}-1}\varpi^{-\frac{1}{4}}e^{-B_{2}\varpi }B_{3}d\varpi +B_{4}\int_{2^{R_{s}^{Th}}-1}^{\infty }\varpi^{-\frac{1}{4}}e^{-(B_{2}+\frac{\sigma_{e}^{2}}{2^{R_{s}^{Th}}})\varpi }B_{3}d\varpi \right), \end{array} \end{array}$$

(33) $$\begin{array}{c} \begin{array}{c} P_{\mathrm{SO}}^{I,T_{2}}=B_{1}\left(\int_{0}^{2^{R_{b}^{Th}}-1}\varpi^{-\frac{1}{4}}e^{-B_{2}\varpi }B_{3}d\varpi +B_{4}\int_{2^{R_{b}^{Th}}-1}^{\infty }\varpi^{-\frac{1}{4}}e^{-(B_{2}+\frac{\sigma_{e}^{2}}{2^{R_{s}^{Th}}})\varpi }B_{3}d\varpi \right), \end{array} \end{array}$$

(34) $$\begin{array}{c} \begin{array}{c} P_{\mathrm{SO}}^{W,T_{1}}=B_{1}\left(\int_{2^{R_{b}^{Th}}-1}^{2^{R_{s}^{Th}}-1}\varpi^{-\frac{1}{4}}e^{-B_{2}\varpi }B_{3}d\varpi +\int_{2^{R_{s}^{Th}}-1}^{\infty }\varpi^{-\frac{1}{4}}e^{-B_{2}\varpi }B_{3}d\varpi \int_{\frac{1+\varpi }{2^{R_{s}^{Th}}}-1}^{\infty }B_{5}e^{-\sigma_{e}^{2}z}dz\right), \end{array} \end{array}$$

(35) $$\begin{array}{c} \begin{array}{c} P_{\mathrm{SO}}^{W,T_{2}}=B_{1}\left(\int_{0}^{2^{R_{b}^{Th}}-1}\varpi^{-\frac{1}{4}}e^{-B_{2}\varpi }B_{3}d\varpi +\int_{2^{R_{b}^{Th}}-1}^{\infty }\varpi^{-\frac{1}{4}}e^{-B_{2}\varpi }B_{3}d\varpi \int_{\frac{1+\varpi }{2^{R_{s}^{Th}}}-1}^{\infty }B_{5}e^{-\sigma_{e}^{2}z}dz\right), \end{array} \end{array}$$

*where, $B_{1}=\frac{4\sigma_{b}^{2}e^{\frac{\pi^{2}N}{2\pi^{2}-32}}}{\left(16-\pi^{2}\right)}\sqrt{\frac{\pi }{N\sigma_{b}}}$, $B_{2}=\frac{8\sigma_{b}^{2}}{(16-\pi^{2})N}$, $B_{3}=I_{-\frac{1}{2}}\left(\frac{4\pi \sigma_{b}\sqrt{\varpi }}{16-\pi^{2}}\right)$, $B_{4}=e^{\sigma_{e}^{2}\left(1-\frac{1}{2^{R_{s}^{Th}}}\right)}$, $B_{5}=\frac{N\sigma_{e}^{2}z+\sigma_{e}^{2}+N}{{\left(Nz+1\right)}^{2}}$.*

**Proof of Theorem** **3.** See Appendix C for proof details. □

#### 4.2.2. GRPA-Based Derivation

Let us define $W_{G}=|\sum_{u=1}^{U}\sum_{t=1}^{A}|g_{t}^{u}\left|\right|h_{t}^{u}|e^{-j[\arg\left(\sum_{t=1}^{A}g_{t}^{u}h_{t}^{u}\right)-∠h_{t}^{u}-∠g_{t}^{u}]}{|}^{2}/\sigma_{b}^{2}$. Similarly to Section 4.1.2, it can be seen that the PDF of SINR achieved with the GRPA-based JPR obeys the Gamma distribution. By employing the Gamma approximation, the associated SINR PDF can be expressed as

(36) $$f_{W}\left(\varpi \right)=\frac{{\left(\frac{b}{\sigma_{b}^{2}}\right)}^{-a}}{\Gamma (a)}\varpi^{a-1}e^{-\frac{\sigma_{b}^{2}\varpi }{b}}.$$

Then, the SOPs with the GRPA-based JPR in different eavesdropping scenarios can be derived in the following theorem.

**Theorem** **4.** *In the scenarios of IWS and WWS, the system SOPs with the GRPA-based JPR strategy can be given by*

(37) $$\begin{array}{c} \begin{array}{c} P_{\mathrm{SO}}^{I,T_{1}}=\int_{0}^{2^{R_{s}^{Th}}-1}B_{6}\varpi^{a-1}e^{-\frac{\sigma_{b}^{2}\varpi }{b}}d\varpi +B_{4}\int_{2^{R_{s}^{Th}}-1}^{\infty }B_{6}\varpi^{a-1}e^{-(\frac{\sigma_{b}^{2}}{b}+\frac{\sigma_{e}^{2}}{2^{R_{s}^{Th}}})\varpi }d\varpi , \end{array} \end{array}$$

(38) $$\begin{array}{c} \begin{array}{c} P_{\mathrm{SO}}^{l,T_{2}}=B_{1}\int_{0}^{2^{R_{b}^{Th}}-1}\varpi^{-\frac{1}{4}}e^{-B_{2}\varpi }B_{3}d\varpi +B_{4}\int_{2^{R_{b}^{Th}}-1}^{\infty }\varpi^{-\frac{1}{4}}e^{-(B_{2}+\frac{\sigma_{e}^{2}}{2^{R_{s}^{Th}}})\varpi }B_{3}d\varpi , \end{array} \end{array}$$

(39) $$\begin{array}{c} \begin{array}{c} P_{\mathrm{SO}}^{W,T_{1}}=\int_{0}^{2^{R_{s}^{Th}}-1}B_{6}\varpi^{a-1}e^{-\frac{\sigma_{b}^{2}\varpi }{b}}d\varpi +\int_{2^{R_{s}^{Th}}-1}^{\infty }B_{6}\varpi^{a-1}e^{-\frac{\sigma_{b}^{2}\varpi }{b}}d\varpi \int_{\frac{1+\varpi }{2^{R_{s}^{Th}}}-1}^{\infty }B_{5}e^{-\sigma_{e}^{2}z}dz, \end{array} \end{array}$$

(40) $$\begin{array}{c} \begin{array}{c} P_{\mathrm{SO}}^{W,T_{2}}=\int_{0}^{2^{R_{b}^{Th}}-1}B_{6}\varpi^{a-1}e^{-\frac{\sigma_{b}^{2}\varpi }{b}}d\varpi +\int_{2^{R_{b}^{Th}}-1}^{\infty }B_{6}\varpi^{a-1}e^{-\frac{\sigma_{b}^{2}\varpi }{b}}d\varpi \int_{\frac{1+\varpi }{2^{R_{s}^{Th}}}-1}^{\infty }B_{5}e^{-\sigma_{e}^{2}z}dz, \end{array} \end{array}$$

*where $B_{6}={\left(\frac{b}{\sigma_{b}^{2}}\right)}^{-a}/\Gamma (a)$.*

**Proof of Theorem** **4.** Similar to the derivation procedure in Theorem 3, the proof is completed by substituting (36) and the PDFs of Eve’s SINRs for different eavesdropping scenarios into (27). □

## 5. Simulation Results

In this section, we illustrate the effectiveness of the proposed ERPA and GRPA-based JPRs in assuring secure SISO transmissions via numerous simulations. Meanwhile, the associated theoretical ESR and SOP results are characterized to verify the correctness of performance derivations. Further, we present one benchmark scheme, namely the IRS-aided secure SISO with random phase shifts, so as to demonstrate the superiority of the proposed secure transmission scheme. Unless otherwise clarified, the specific simulation parameters are given in the figures.

**Figure 3:**
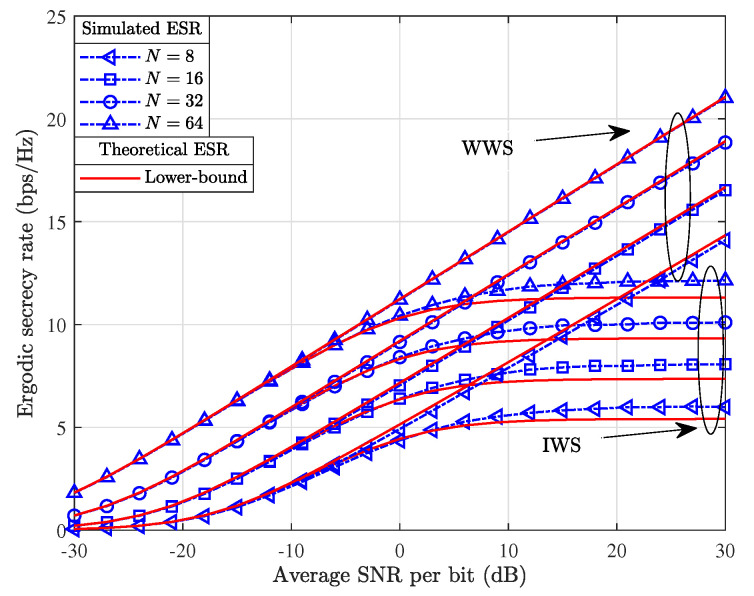
Comparison of simulated and theoretical results for the achievable ESR with the ERPA-based JPR scheme.

Figure 3 depicts the simulated and theoretical ESR results of the proposed SISO secure transmissions with the ERPA-based JPR strategy when N∈{8,16,32,64}. From Figure 3, it can be seen that the achievable ESR levels off as the average SNR increases to the investigated high-SNR region in the scenario of IWS, where Eve has no transmission connection with the IRS. Moreover, the ESR achieved in the IWS is lower than that in the WWS, which verifies that the eavesdropping IWS scenario is the worst-case scenario for BS. However, even in the worst-case scenario for BS, the ESR achieved with the ERPA-based JPR scheme elevated rapidly in the low-to-medium SNR region, which demonstrates the effectiveness of the proposed ERPA-based secure transmission. Second, the simulated and theoretical ESR results fit tightly with each other upon utilizing the ERPA-based JPR in the scenario of WWS, where Eve has a transmission link with the IRS. Moreover, it is worth noting that for both the IWS and WWS, the accuracy of the theoretical ESR decreases as *N* is selected small. This is because the theoretical lower bound of ESR is derived via CLT-based approximation, rendering that *N* is selected to be large enough. Finally, both the simulated and theoretical ESR increase significantly as *N* increases. This is because there is an increasing number of multipath links to improve the SNR at BS when *N* increases from 8 to 64.

**Figure 4:**
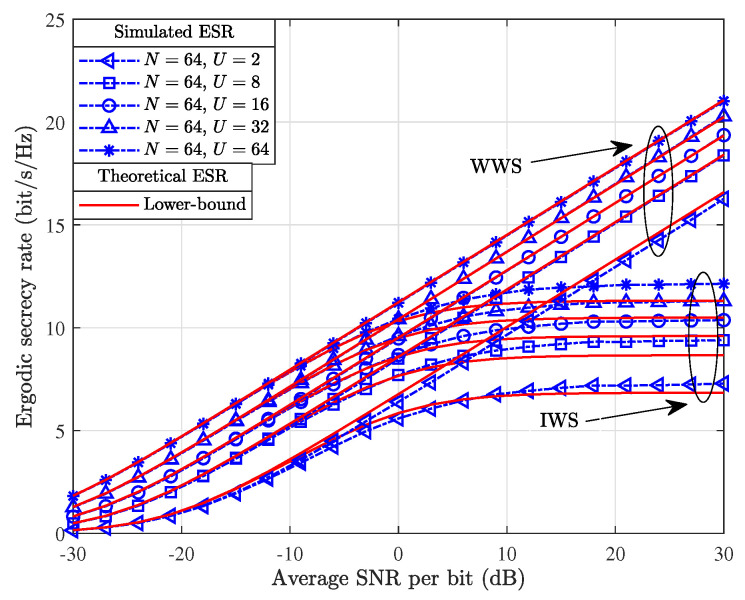
Comparison of simulatied and theoretical results for the achievable ESR with the GRPA-based JPR scheme.

Figure 4 demonstrates the simulated and theoretical ESR results achieved by utilizing the GRPA-based JPR with U∈{2,8,16,64} and N=64. It is worth noting that given N=64, the GRPA-based JPR with U=64 is actually the ERPA-based JPR. From Figure 4, we can observe that in the WWS scenario, the achievable ESR increases almost linearly over a wide range of SNR. We can obtain that as *U* increases, the gap between the simulated and theoretical ESR results achieved upon employing the GRPA and ERPA-based JPRs become smaller and smaller. This further exemplifies that the GRPA-based JPR can not only reduce the system implementation complexity but also ensure a certain high-level of security. In addition, Figure 4 also characterizes the theoretical ESR results obtained by employing the Gamma approximation, which are compared with the simulated ESR results to validate the accuracy of the closed-form ESR derivations.

**Figure 5:**
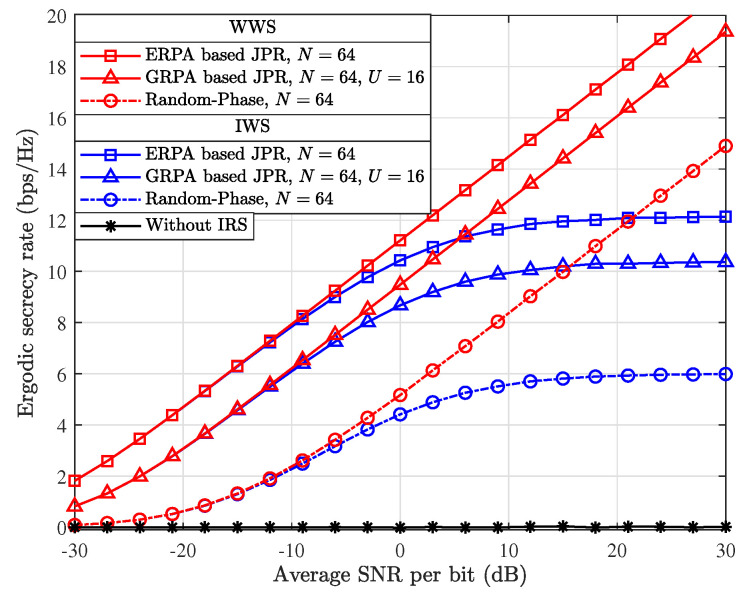
Comparison of simulated ESR results with different secure transmission schemes.

Figure 5 presents a comprehensive comparison of the simulated ESR results, which are achieved by utilizing different SISO secure transmission schemes, such as the ERPA and GRPA-based JPRs, IRS-aided secure SISO with random phase shifts (random phase), and without the assistance of IRS (without IRS). As can be observed from Figure 5, the ERPA-based JPR can outperform the random-phase scheme in terms of ESR performance. This superiority is obtained due to the complete cancellation of the perturbed phase at BS, and hence the maximum SNR is attained when the ERPA-based JPR is exploited. In addition, it is worth noting that the GRPA-based JPR also outperforms the random-phase scheme in the perspective of simulated ESR. This demonstrates the significant advantage of GRPA-based JPR in maintaining an acceptable level of secure performance while reducing the system implementation complexity. Furthermore, it can be observed that the ESR of the scheme without IRS is almost 0, which is significantly lower than that of the schemes with the assistance of IRS. This is because the IRS can be utilized for the adaptive adjustment of a wireless propagation channel and hence for enhancing the system’s transmission rate and secrecy performance remarkably. Even with a random adjustment of IRS phase shifts, the SISO transmission system is capable of achieving with a substantially enhanced ESR. Overall, the comparative analysis presented in Figure 5 not only verifies the effectiveness of our proposed strategies but also embodies the importance of properly adjusting the IRS reflection in enhancing the system secrecy.

**Figure 6:**
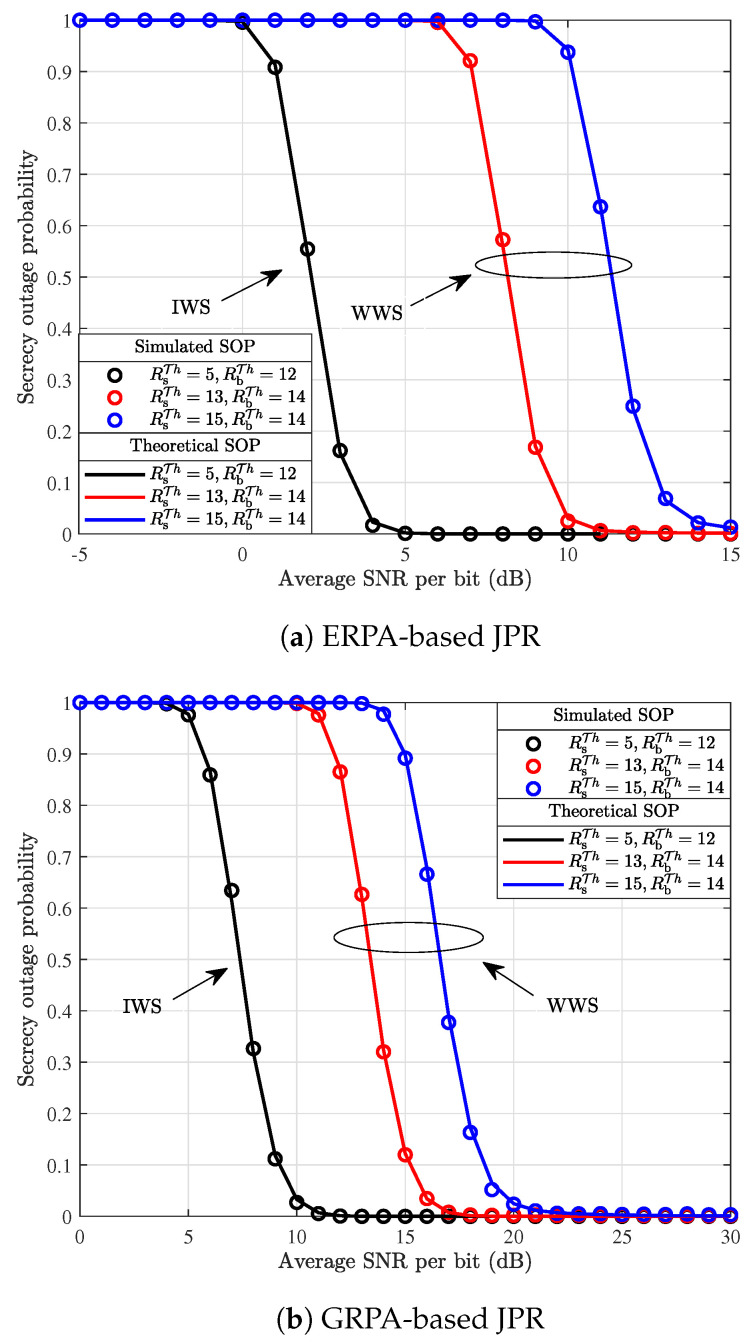
Comparison of simulated and theoretical SOP results with different JPR schemes.

Figure 6 illustrates a comparison of simulated and theoretical SOP results achieved with the ERPA and GRPA-based JPR schemes, respectively. From Figure 6, it is intuitive to observe that the theoretical SOP results are consistent with the corresponding simulated performance results, while in general, the SOP performance decreases as the average SNR increases. In addition, the average SNR required for the SOP results decreasing to 0 is smaller in the IWS scenario than that in the WWS scenario. In the scenario of WWS, by letting $R_{s}^{Th}=5$ bps/Hz and $R_{b}^{Th}=12$ bps/Hz, the SOP with the ERPA-based JPR leads the fastest descent rate, while the SOP decreases to 0 at about SNR=5 dB. Moreover, given the selected rate thresholds, the system SOP with the ERPA-based JPR surpasses that with the GRPA-based JPR. In addition, given the same transmission rate threshold at BS, the average SNR required for the decreasing of SOP to 0 increases gradually as the secrecy rate threshold increases.

**Figure 7:**
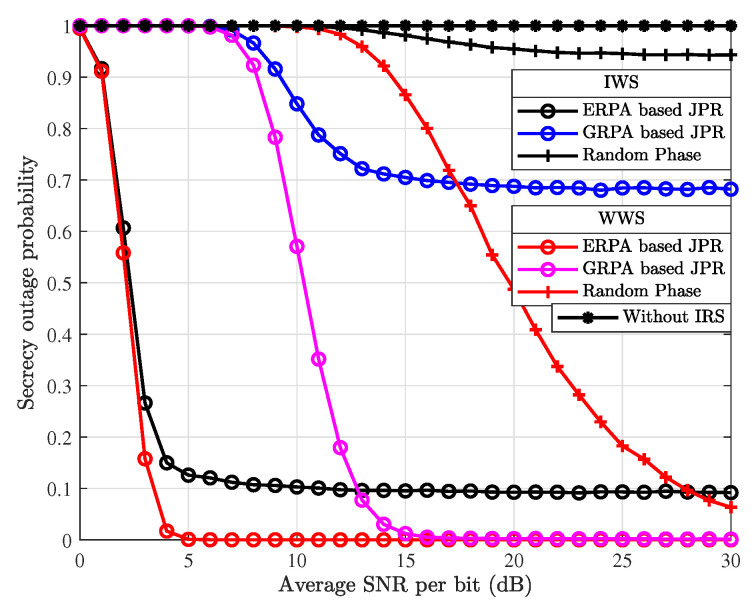
Comparison of simulated SOP results with different secure transmission schemes.

Figure 7 characterizes the simulated SOP performance results, which are obtained by using four different secure transmission schemes with parameter settings of $R_{s}^{Th}=10$ bps/Hz and $R_{b}^{Th}=12$ bps/Hz. As can be seen from Figure 7, the SOP performance with the ERPA and GRPA-based JPR schemes are much better than that of the random phase and without IRS schemes. It can also be observed that when considering the IWS scenario and employing the random phase scheme, the system transmission is interrupted with high probability. In addition, the SOP achieved with the ERPA-based JPR is lower than that with the GRPA-based JPR. The system SOP performance achieved in the WWS scenario is better than that in the IWS scenario. This intuitively substantiates the secrecy superiority that can be attained in the WWS scenario. Meanwhile, the comparative analysis achieved from Figure 7 also illustrates the necessity of rational design of the IRS phase shifts in attaining ideally enhanced system secrecy.

## 6. Conclusions

In this paper, an IRS-based SISO secure transmission system has been investigated. In order to significantly enhance the security of the system, we have introduced an JPR-based transmission mechanism, where a random perturbation factor at the transmitter side and a class of RPA scheme at the IRS are accomplished, such that the negative phase effect at the legitimate receiver can be well counteracted. In addition, based on the IRS element grouping design, both the ERPA and GRPA-based JPRs have been proposed to achieve with different trade-offs between the system security performance and implementation complexity, respectively. We have also proposed two eavesdropping scenarios based on the occasional connection situations between the IRS and Eve. Given that the ERPA and GRPA-based JPRs are utilized, the ESR and SOP results that system achieves under different eavesdropping scenarios have been analytically deduced. Simulation results demonstrate that the proposed RPA-based JPR can effectively and comprehensively guarantee the security of SISO transmission without additional power and hardware consumptions.

## Data Availability

The data used to support the findings of this study are included in the article.

## Appendix A

To begin with, we have the PDF of the non-central chi-square distribution as follows.

(A1) $$f\left(x;k,\lambda \right)=\frac{1}{2\sigma^{2}}e^{-\frac{x+\lambda }{2\sigma^{2}}}{\left(\frac{x}{\lambda }\right)}^{\frac{k}{4}-\frac{1}{2}}I_{\frac{k}{2}-1}\left(\frac{\sqrt{\lambda x}}{\sigma^{2}}\right),x\geq 0,$$

here, *k* is the degrees of freedom, $\lambda =\sum_{i=1}^{k}{\mu_{i}}^{2}$, where $\mu_{i}$ is the mean of the i-th superimposed variable. Then, the expectation of $|g\Phi h{|}^{2}$ can be deduced as

(A2) $$\begin{array}{c} \begin{array}{cc} E_{g\Phi h}\left[|g\Phi h{|}^{2}\right] & =\int_{0}^{\infty }xf(x;1,\frac{\pi^{2}}{16}N^{2})dx\\ \; & \overset{a}{=}\int_{0}^{\infty }x\frac{1}{2\times \frac{16-\pi^{2}}{16}N}e^{-\frac{x+\frac{\pi^{2}}{16}N^{2}}{2\times \frac{16-\pi^{2}}{16}N}}{\left(\frac{x}{\frac{\pi^{2}}{16}N^{2}}\right)}^{-\frac{1}{4}}I_{-\frac{1}{2}}\left(\frac{\sqrt{\frac{\pi^{2}}{16}N^{2}x}}{\frac{16-\pi^{2}}{16}N}\right)dx\\ \; & \overset{b}{=}\frac{4e^{\frac{\pi^{2}N}{2\pi^{2}-32}}}{16-\pi^{2}}\sqrt{\frac{\pi }{N}}\frac{\Gamma (\frac{3}{2})}{\frac{2\pi }{16-\pi^{2}}\Gamma \left(\frac{1}{2}\right)}e^{\frac{\pi^{2}N}{4(16-\pi^{2})}}{\left(\frac{8}{(16-\pi^{2})N}\right)}^{-\frac{5}{4}}M_{-\frac{5}{4},-\frac{1}{4}}\left(\frac{\pi^{2}N}{2(16-\pi^{2})}\right)\\ \; & =\frac{e^{\frac{\pi^{2}N}{4\pi^{2}-64}}}{\sqrt{\pi N}}{\left(\frac{8}{(16-\pi^{2})N}\right)}^{-\frac{5}{4}}M_{-\frac{5}{4},-\frac{1}{4}}\left(\frac{\pi^{2}N}{2(16-\pi^{2})}\right), \end{array} \end{array}$$

where equality ‘a’ is obtained by substituting the expectation $\lambda =\frac{\pi }{4}N$ and the variance $\sigma =\frac{16-\pi^{2}}{16}N$ of *x* into (A1), while equality ‘b’ is obtained by utilizing formula (6.643.2) in the integral table [37].

## Appendix B

Based on (25), we can approximate the PDF of $x=|g\Phi h{|}^{2}$ with the assistance of the Gamma approximation as

(A3) $$f\left(x\right)=\frac{x^{a-1}}{\Gamma (a)}b^{-a}e^{-\frac{x}{b}},$$

in which

(A4) $$\begin{array}{c} a={\left(E\left[x\right]\right)}^{2}/E\left[{\left(x-E\left[x\right]\right)}^{2}\right],\\ b=(E[{\left(x-E\left[x\right]\right)}^{2}/E\left[x\right], \end{array}$$

where *a* and *b* are the shape and scale parameters, separately. Since a,b are functions of variable *x*, it is complicated to derive their analytical results directly. Whereas, according to the discussion in the literature [38], the associated Gamma distribution parameters can be obtained by about $10^{4}$ Monte Carlo simulations. Eventually, the expectation of *x* can be obtained as

(A5) $$E_{g\Phi h}\left[x\right]=\int_{0}^{\infty }x^{a}\frac{b^{-a}}{\Gamma (a)}e^{-\frac{x}{b}}dx.$$

Substituting (A5) into (17), the closed-form lower bound on ${\bar{R}}_{b}$ can be obtained as

(A6) $$\begin{array}{ccc} {\bar{R}}_{b} & \geq \log_{2}\left(1+\frac{\int_{0}^{\infty }x^{a}\frac{b^{-a}}{\Gamma (a)}e^{-\frac{x}{b}}dx}{\sigma_{b}^{2}}\right)\; & =\log_{2}\left(1+\frac{b\Gamma (a+1)}{\Gamma \left(a\right)\sigma_{b}^{2}}\right) \end{array}.$$

## Appendix C

In the scenarios of IWS and WWS, the PDF of the SNR at BS with the ERPA-based JPR is the same as that in (18). Upon substituting the PDFs of $\gamma_{b}$ and $\gamma_{e}$ into (27), in the scenario of IWS, the approximate semi-closed-form SOP derivations for the cases of $T_{1}$ and $T_{2}$ are detailed as follows. First, the SOP achieved for the case of $T_{1}$ can be deduced as

(A7) $$\begin{array}{c} \begin{array}{cc} P_{\mathrm{SO}}^{I,T_{1}} & =\int_{0}^{2^{R_{s}^{Th}}-1}f(\varpi ;1,\frac{\pi^{2}}{16\sigma_{b}^{2}}N^{2})d\varpi +\int_{2^{R_{s}^{Th}}-1}^{\infty }f(\varpi ;1,\frac{\pi^{2}}{16\sigma_{b}^{2}}N^{2})d\varpi \int_{\frac{1+\varpi }{2^{R_{s}^{Th}}}-1}^{\infty }f_{\Lambda }\left(v\right)dv\\ \; & \overset{a}{=}\int_{0}^{2^{R_{s}^{Th}}-1}B_{2}e^{-B_{2}\left(\varpi +\frac{\pi^{2}}{16\sigma_{b}^{2}}N^{2}\right)}{\left(\frac{\varpi }{\frac{\pi^{2}}{16\sigma_{b}^{2}}N^{2}}\right)}^{-\frac{1}{4}}I_{-\frac{1}{2}}\left(\frac{\sqrt{\frac{\pi^{2}}{16\sigma_{b}^{2}}N^{2}\varpi }}{\frac{16-\pi^{2}}{16\sigma_{b}^{2}}N}\right)d\varpi \\ \; & \;+\int_{2^{R_{s}^{Th}}-1}^{\infty }B_{2}e^{-B_{2}\left(\varpi +\frac{\pi^{2}}{16\sigma_{b}^{2}}N^{2}\right)}{\left(\frac{\varpi }{\frac{\pi^{2}}{16\sigma_{b}^{2}}N^{2}}\right)}^{-\frac{1}{4}}I_{-\frac{1}{2}}\left(\frac{\sqrt{\frac{\pi^{2}}{16\sigma_{b}^{2}}N^{2}\varpi }}{\frac{16-\pi^{2}}{16\sigma_{b}^{2}}N}\right)d\varpi \int_{\frac{1+\varpi }{2^{R_{s}^{Th}}}-1}^{\infty }\sigma_{e}^{2}e^{-\sigma_{e}^{2}v}dv\\ \; & =B_{1}\left(\int_{2^{R_{b}^{Th}}-1}^{2^{R_{s}^{Th}}-1}\varpi^{-\frac{1}{4}}e^{-B_{2}\varpi }B_{3}d\varpi +B_{4}\int_{2^{R_{s}^{Th}}-1}^{\infty }\varpi^{-\frac{1}{4}}e^{-(B_{2}+\frac{\sigma_{e}^{2}}{2^{R_{s}^{Th}}})\varpi }B_{3}d\varpi \right). \end{array} \end{array}$$

Similarly, we can obtain the SOP achieved for the case of $T_{2}$ as

(A8) $$\begin{array}{c} \begin{array}{c} P_{\mathrm{SO}}^{I,T_{2}}=B_{1}\left(\int_{0}^{2^{R_{b}^{Th}}-1}\varpi^{-\frac{1}{4}}e^{-B_{2}\varpi }B_{3}d\varpi +B_{4}\int_{2^{R_{b}^{Th}}-1}^{\infty }\varpi^{-\frac{1}{4}}e^{-(B_{2}+\frac{\sigma_{e}^{2}}{2^{R_{s}^{Th}}})\varpi }B_{3}d\varpi \right). \end{array} \end{array}$$

Then, in the scenario of WWS, we also derive the approximate semi-closed-form SOPs in associated with the two cases of $T_{1}$ and $T_{2}$ as

(A9) $$\begin{array}{c} \begin{array}{c} P_{\mathrm{SO}}^{W,T_{1}}=B_{1}\left(\int_{2^{R_{b}^{Th}}-1}^{2^{R_{s}^{Th}}-1}\varpi^{-\frac{1}{4}}e^{-B_{2}\varpi }B_{3}d\varpi +\int_{2^{R_{s}^{Th}}-1}^{\infty }\varpi^{-\frac{1}{4}}e^{-B_{2}\varpi }B_{3}d\varpi \int_{\frac{1+\varpi }{2^{R_{s}^{Th}}}-1}^{\infty }B_{5}e^{-\sigma_{e}^{2}z}dz\right), \end{array} \end{array}$$

(A10) $$\begin{array}{c} \begin{array}{c} P_{\mathrm{SO}}^{W,T_{2}}=B_{1}\left(\int_{0}^{2^{R_{b}^{Th}}-1}\varpi^{-\frac{1}{4}}e^{-B_{2}\varpi }B_{3}d\varpi +\int_{2^{R_{b}^{Th}}-1}^{\infty }\varpi^{-\frac{1}{4}}e^{-B_{2}\varpi }B_{3}d\varpi \int_{\frac{1+\varpi }{2^{R_{s}^{Th}}}-1}^{\infty }B_{5}e^{-\sigma_{e}^{2}z}dz\right). \end{array} \end{array}$$
